# Voices from the Margins: Barriers and Facilitators to HPV Self-Sampling Among Structurally Marginalized People with a Cervix in the Greater Toronto Area and Ontario

**DOI:** 10.3390/curroncol32060327

**Published:** 2025-06-03

**Authors:** Mandana Vahabi, Natasha Kithulegoda, Masoomeh Moosapoor, Aisha Lofters, Josephine Pui-Hing Wong, Abdolreza Akbarian, Jenna Hynes

**Affiliations:** 1 Lawrence Bloomberg Faculty of Nursing, University of Toronto, Toronto, ON M5T 1P8, Canada; 2 MAP Centre for Urban Health Solutions, Li Ka Shing Knowledge Institute, St. Michael’s Hospital, Toronto, ON M5B 1W8, Canada; 3 Institute for Clinical Evaluative Sciences, Toronto, ON M4N 3M5, Canada; aisha.lofters@wchospital.ca; 4 Daphne Cockwell School of Nursing, Toronto Metropolitan University, Toronto, ON M5B 2K3, Canada; masoomeh.moosapoor@torontomu.ca (M.M.); jph.wong@torontomu.ca (J.P.-H.W.); aakbarian@torontomu.ca (A.A.); 5 Institute for Health Policy, Management and Evaluation, University of Toronto, Toronto, ON M5T 3M7, Canada; natasha.kithulegoda@gmail.com; 6 Peter Gilgan Centre for Women’s Cancers, Women’s College Hospital, Toronto, ON M5S 1B2, Canada; 7 Dalla Lana School of Public Health, Toronto, ON M5T 3M7, Canada; 8 Maggie’s Toronto, Toronto, ON M3C 0C3, Canada

**Keywords:** cervical cancer, screening, HPV self-sampling, sex workers, formerly incarcerated individuals, access to healthcare, discrimination, trauma-informed care

## Abstract

Sex workers and formerly incarcerated people with a cervix face significant structural, interpersonal, and emotional barriers to cervical cancer screening, despite being at elevated risk for HPV infection. HPV self-sampling (HPV-SS) is a validated, user-directed method that has the potential to address these barriers, yet it remains excluded from Ontario’s organized screening program. This qualitative study explored the lived experiences of structurally marginalized individuals with a cervix who were offered HPV-SS as part of a mixed-methods pilot in the Greater Toronto Area. Five virtual focus groups were conducted with 34 participants, including both those who used the HPV-SS kit and those who declined it. Using inductive thematic analysis, we identified barriers to traditional screening including fear, stigma, mistrust of healthcare providers, logistical constraints, and a lack of accessible information. HPV-SS was widely described as more acceptable, empowering, and emotionally manageable, offering participants autonomy, privacy, and control over their care. Concerns included swab design, uncertainty about correct use, and unclear follow-up after positive results. Participants offered concrete, community-informed recommendations to improve HPV-SS implementation, including opt-in distribution models, gender-affirming language, and trauma-informed educational materials. The findings highlight the urgent need to integrate HPV-SS into organized screening programs to advance equitable access to cervical cancer prevention for marginalized populations.

## 1. Introduction

Sex workers and formerly incarcerated women are among the structurally marginalized groups who—despite being at heightened risk for HPV infection (the primary cause of cervical cancer)—remain severely under-screened [1,2,3,4,5]. Barriers to cervical cancer screening in these populations are multifaceted, including limited awareness of HPV and its link to cancer, experiences of stigma and discrimination, a lack of access to consistent healthcare providers, logistical challenges (e.g., transportation, clinic hours, childcare), and cultural taboos surrounding both their social identities and sexual health [6,7,8,9,10,11].

In Spring 2025, Ontario’s organized screening program transitioned from Pap tests to high-risk HPV testing as the primary screening modality. However, HPV self-sampling (HPV-SS)—a validated, user-directed method that enables individuals to collect their own vaginal specimens privately—has not yet been incorporated into the program [12]. This exclusion persists despite strong evidence demonstrating HPV-SS’s accuracy, acceptability, and potential to improve screening rates among diverse under-screened populations [13,14,15,16], including its integration into the provincial cervical cancer screening program in British Columbia and pilots conducted with under- or never-screened women in Manitoba and Ontario [17,18,19,20].

Given the persistently low cervical cancer screening rates among structurally marginalized groups such as sex workers and formerly incarcerated women, it is critical to evaluate the feasibility and acceptability of evidence-based strategies like HPV-SS. Traditional screening methods, such as the Pap test, and newer approaches like clinician-collected HPV testing both require a pelvic examination with a speculum [14]—a procedure that many individuals find invasive, uncomfortable, and retraumatizing, particularly for those with histories of stigma, discrimination, or trauma [21]. By allowing individuals to self-collect vaginal specimens without a speculum, HPV-SS empowers participants and addresses key structural and psychosocial obstacles to screening.

To address this gap, we conducted a community-based, mixed-methods pilot study in the Greater Toronto Area (GTA). We recruited 84 sex workers and formerly incarcerated women with a cervix who were under-screened or never screened for cervical cancer and offered them HPV-SS as an alternative screening method. The quantitative phase of this study demonstrated the high uptake and feasibility of HPV-SS and has been published in *Current Oncology* (“Breaking Barriers: Empowering Cervical Cancer Screening with HPV Self-Sampling for Sex Workers and Formerly Incarcerated Women in Toronto”) [22]. Building on these results, the qualitative phase engaged approximately 40% of participants (n = 34) in focus groups to explore their lived experiences with HPV-SS and to elicit their perspectives on the facilitators and barriers to its use.

This paper focuses on the findings of the study’s qualitative component, shedding light on the specific challenges (e.g., mistrust of healthcare institutions, fear of judgment, uncertainty about self-sampling technique) and facilitators (e.g., privacy, empowerment, flexible access) that influence HPV-SS uptake among sex workers and formerly incarcerated women. Our findings underscore the urgent need to integrate HPV self-sampling into organized screening programs to ensure equitable access to cervical cancer prevention for marginalized communities.

## 2. Methods

### 2.1. Study Design and Recruitment

The qualitative component of our community-based mixed-methods pilot study consisted of five virtual focus groups conducted with a total of 34 participants residing in the Greater Toronto Area (GTA). Participants were individuals with a cervix, who identified as sex workers and/or formerly incarcerated people. Eligibility criteria included the following: self-identifying as a sex worker or formerly incarcerated; being aged 25 to 69 years; having a cervix; reporting more than four years since their last Pap test (including no history of Pap test); having a history of sexual activity; residing in the GTA; being able to communicate in English; providing informed consent; and willingness to share contact information with the research team. Individuals with a history of hysterectomy were excluded.

**Figure 1:**
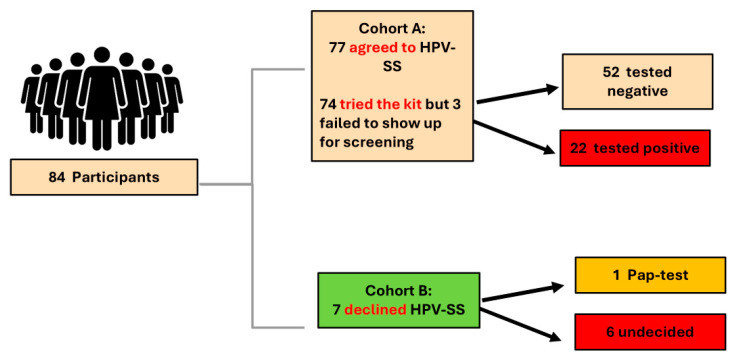
Eligible participant flow diagram.

Participants were recruited from those who had previously completed the study’s quantitative survey [22]. Eligible participants were divided into two cohorts: Cohort A, consisting of women who agreed to use the HPV-SS kit, and Cohort B, consisting of those who declined self-sampling (see Figure 1). Those who declined HPV-SS did not receive any further testing as part of this study. During survey participation, women were invited to participate in a follow-up focus group. Those who expressed interest were subsequently contacted by the study’s Research Associate to confirm eligibility and obtain informed consent.

Three focus groups were conducted with Cohort A participants to explore their experiences with HPV self-sampling (HPV-SS), including one focus group specifically with women who had received a positive HPV test result. Two additional focus groups were held with Cohort B participants to understand their reasons for declining self-sampling and their attitudes toward cervical cancer screening. Each focus group included two to nine participants and lasted approximately two hours, including 10 min breaks. All sessions were conducted virtually via Zoom.

To minimize barriers to participation, private rooms and computer access were made available at the collaborating community centers (Maggie’s Toronto Sex Workers Action Project and PASAN) for participants who did not have access to appropriate technology. All participants received a CAD 50 honorarium for their time and to offset potential internet-related costs. The study was reviewed and approved by the Research Ethics Boards of Toronto Metropolitan University (REB #2023-165) and the University of Toronto (REB #45488).

### 2.2. Data Collection

Focus groups were facilitated in English by members of the research team (NK, AA, MM) using a semi-structured focus group guide. The guide included questions exploring participants’ knowledge and attitudes toward cervical cancer screening, perceived barriers and facilitators to HPV-SS, and recommendations for improving access to and the uptake of cervical cancer screening among marginalized populations. For participants who had tested positive for HPV, additional questions focused on their follow-up care experiences.

Each focus group was audio-recorded with participants’ consent and transcribed verbatim by two members of the research team (AA, MM). To maintain confidentiality, participants were reminded at the beginning of each session that although the research team would protect their privacy, they could not guarantee that other participants would not disclose information shared during the group discussion. Participants were encouraged to share only information they felt comfortable discussing in a group setting and used a pseudonym if preferred. We did not systematically collect data during the qualitative phase of this work on whether participants identified as sex workers, formerly incarcerated, or both. This decision was made to prioritize participant confidentiality, particularly given the small sample size and potential overlap of identities. Thus, individual quotes are not attributed to specific identities in the findings.

### 2.3. Data Analysis

All transcripts were analyzed using an inductive thematic analysis approach. A member of the research team who has formal training in qualitative research (NK) conducted the open coding of the transcripts on NVivo 14. Codes were organized into emerging themes that reflected participants’ experiences, perceived barriers and facilitators to HPV-SS, and suggestions for improving cervical cancer screening practices. The coding process and thematic development were reviewed collaboratively by the broader research team to ensure accuracy, consistency, and rigor. This study was designed and reported in alignment with the COREQ checklist.

## 3. Results

A total of 34 participants, consisting of sex workers and formerly incarcerated people who have a cervix, participated in five focus groups. A total of 14 participants were recruited through PASAN, an organization that advocates for individuals who are incarcerated or formerly incarcerated, and 20 participants were recruited through Maggie’s Toronto Sex Workers Action Project. The focus groups were conducted via Zoom and lasted approximately 90 min each. The findings are presented under four overarching themes: Barriers to Cervical Cancer Screening, Facilitators of HPV-SS, Barriers to HPV-SS, and Suggestions for Future HPV-SS Implementation.

### 3.1. Barriers to Cervical Cancer Screening

Participants shared a range of personal, interpersonal, and systemic challenges that shaped their access to and experiences with cervical cancer screening. These barriers were often intertwined with experiences of stigma fear and perceptions of exclusion within healthcare settings.

#### 3.1.1. Fear and Stigma

Participants identified multiple barriers to cervical cancer screening, with negative experiences in clinical settings emerging as a prominent theme. A common concern was the unpredictability of provider behavior. “*Every time we have to go into a clinic or see a doctor deal with these issues. It’s also like…like you, you get to avoid a lot of trauma…you know, like, because every doctor’s visit, especially if you’re dealing with stuff below the [belly]…It’s like, it’s a it’s a potential trauma…It’s one of the things that you kind of have to brace yourself to do, or at least I do cause you never know how it’s gonna go. Who? What doctor you’re gonna get…*” (P02). Participants reported being treated disrespectfully and receiving inadequate information: “*You can get really disrespected like I’ve had people being like, very, very disregarding to me.*” (P01).

Participants described a pervasive fear surrounding cervical cancer and screening. For many, early interactions with healthcare providers instilled long-lasting anxiety and a sense of fatalism. One participant described an experience with a physician making them feel “*If I get it, my life’s just screwed. That would just be the end of things for me.*” (P03). Fear was often rooted in personal trauma, and some participants associated cervical cancer with death: “*…I’m saying that to me it just symbolizes something like a death sentence. You know. When I hear of cervical cancer, the next thing that comes to my brain is death.*” (P18).

Fear and stigma were consistently cited as significant deterrents to screening. Several participants articulated how their identities as sex workers, queer individuals, or trans people intersected with healthcare provider bias. Past negative experiences with healthcare providers, particularly those involving stigma associated with sex work, influenced participants’ willingness to engage with providers. “*Having been a sex worker, just like…like doctors like kind, like use like use HPV, Cervical cancer, STDs to like scare you, scare you into like a more moral line of work…I’ve never come across like somebody who’s…you know, like had like a helpful…like practitioner…like in that realm. So it’s just. It’s frustrating. Disappointing…makes me mad.*” (P02). Overall, participants reported a general stigma among healthcare providers when they sought care: “*Doctors will gaslight you… because they don’t believe in sex work.*” (P18).

Some participants described healthcare providers’ communication styles as fear-inducing. “They told me they were like, you have a 75 to 80% chance of getting cancer…I was just in a completely like scared mindset… Sometimes it feels like…I don’t know. I don’t know (closes eyes) I just like I just want to add on to that that I the fear the fear, like the fear from doctors.” (P04). Others described encounters with providers that felt invasive or judgmental: “I can’t remember exactly what it was, but the physician I went to see was a very old man…And well, he kinda like, yeah scared tactic as well. He was like…asking a little too many questions I think about like my sex…practice life…all of it…” (P01).

Beyond fear and stigma, several participants described feeling underinformed or misinformed about cervical cancer and screening options. This lack of accessible, relevant information was identified as a critical barrier to seeking care and participating in screening.

#### 3.1.2. Education and Access to Information

Participants described limited access to clear, accurate, and personally relevant information about cervical cancer and HPV-SS. Across all focus groups, participants highlighted that education was either missing or insufficient and made it difficult to make informed decisions. One participant stated “*All those things… we definitely don’t receive enough education… there’s definitely not enough information out there… that you don’t have to kind of like carve around to find*” (P01). Another participant noted that their profession warranted additional attention that was never given: “*There’s a bunch of things that can happen to my body from having sex and doing sex work. The problem I have is, I have no breakdown of what those things look like physically and verbally, because people have taboo around that, so I can’t access those things.*” (P11). Many participants noted that without accessible information about sexual health, cervical cancer, and screening, decision-making around care can be difficult: “*And so I think it’s the lack of information that’s scariest to me, because I don’t know how to make an informed decision without the information…And then also, just wanna second, like it’s it feels really scary.*” (P13). These experiences highlight the inaccessibility of information for sex worker and formerly incarcerated populations, and a general lack of knowledge about sexual health, cervical cancer, and screening.

In addition to informational gaps, participants outlined significant structural obstacles that hindered timely and equitable access to screening. These challenges ranged from healthcare system delays to geographic and logistical limitations.

#### 3.1.3. Structural Barriers

Access to screening was shaped by logistical and structural barriers, such as long wait times, limited physician availability, and perceptions of unsupportive healthcare environments. One participant stated the following: “*I think a walk-in clinic can do for me better, because if you are to see a personal doctor, you must book an appointment like I was to see again a doctor. I have not yet been able to see for the last 6 months. I’m still on a waiting list.*” (P22). A lack of physician follow-up was described: “*My doctor hasn’t spoken to me since COVID… my health card’s expired*.” (P12). Additionally, several participants expressed hesitancy around seeking care: “*Sometimes the trauma of even booking an appointment is enough to stop me from trying.*” (P11). Transportation issues and challenges faced by those living in rural or remote areas were also noted: “*People have to work and people who do work often find that the hours that they have to work. They have to take off to go to a doctor’s appointment. And so that’s not always feasible. I think about younger generation who don’t want to let their parents know or don’t want to make their family doctors know.*” (P08).

### 3.2. Facilitators of HPV-SS

Despite these challenges, participants also articulated several facilitators that made HPV-SS (HPV-SS) more accessible, empowering, and emotionally manageable than traditional screening methods.

#### 3.2.1. Empowerment and Autonomy

Participants identified several facilitators that increased their willingness to engage with HPV-SS. Many participants described HPV-SS as giving them the ability to reclaim control, and avoid stigma and medical trauma: “*It gives you control back into your health and your healthcare.*” (P02); “*Only your hands are on you through the whole process.*” (P13). Participants felt that they had more control over the test versus testing with a healthcare provider: “*So being able to like…that like have getting that control, that freedom, that privacy back is…is for me is probably one of the biggest…one of the biggest like…like pros about the self-test. For me personally.*” (P02). Privacy was frequently described as a facilitator, as it minimized potentially traumatic provider interactions: “*I also felt so much more comfortable than going to the doctors I’ve also experienced, like just trauma of being on a table and having a doctor administrate a Pap test for you, it’s a lot more uncomfortable than being in a public washroom, and it felt like a covid test, but for your buzzes, so that like felt pretty easy and pretty doable*” (P21). The ability to participate in HPV-SS without disclosure of profession, gender identity, or trauma history was especially empowering.

The autonomy offered by HPV-SS was particularly significant for those with described histories of trauma. Participants emphasized how conducting the test in a discreet and safe environment reduced the emotional labor and fear associated with traditional screenings. “*I’m like two spirits, and like trans. So, it was like kind of scary at first and like I’ve definitely like gone for Pap smears with like a doctor before that kind of like made me feel kind of like shame, for like while I was doing the test as opposed to like, you guys were like so friendly and like, discreet about it all, like it definitely felt like, safe. And yeah, like, I definitely was like, happy to like, go through with it, you know, like, I think it’s important.*” (P29).

In addition to the psychological and emotional benefits of privacy and autonomy, participants praised the HPV-SS method for its simplicity and overall user-friendliness.

#### 3.2.2. Ease of Use

Overall, participants expressed overwhelming appreciation of HPV-SS as a method for cervical cancer screening. HPV-SS was viewed as an easy-to-use, user-friendly, and convenient method of screening: “*Well, in one word, it was user friendly and very explanatory. And it’s like, it’s okay. It’s not hurtful. Unlike the Pap test I had yesterday that one was kind of crampy. So, it’s user friendly. I see that.*” (P32). “*I really liked the self-sampling. It made things very easy and accessible; seem over easy, a lot; easier than a covid swab.*” (P14). A few participants described the feeling of doing the procedure incorrectly, due to its simplicity and ease of use: “*…it was so easy that I was like, did I do something wrong? Have I like swabbed in that like I don’t it? Just it felt I was like, is that really, truly it like, am I good? Could? I don’t know. I’m not a doctor, could I mess this up somehow? But the instructions were so simple and straightforward that I was like, Okay, like, I reread them. I can’t be missing anything. And so I felt good about that…*” (P03). Many participants described HPV-SS as accessible and minimally invasive: “*The experience wasn’t…it was less awful than like…putting a tampon in for the 1st time…as like a point of reference for discomfort.*” (P07).

### 3.3. Barriers to HPV-SS

While self-sampling was largely supported by participants, some participants expressed concerns regarding the HPV-SS swab and follow-up after a positive result. 

#### Concerns About HPV-SS and Follow-Up

The flimsiness of the swab itself was raised as a concern in one focus group, particularly for participants with larger bodies: “*Trying to get something flimsy into a hole you can’t see without touching it is really difficult… It’s like trying to… Still, you know, like an Al Dente like noodle like spaghetti noodle. It’s kinda hard to, I don’t know, that’s the best metaphor I can think of.*” (P02). Participants who had positive results expressed a great desire for clarity in follow-up procedures after a positive result. The absence of clear instructions on how to interpret results was evident: “*Okay, I just, I think I also wanted to say that I think I would have definitely needed some kind of like 1–800 number as a right away…*” (P03). Another participant stated that no clear information was given at their follow-up procedure, which created overall discomfort: “*So that’s why I go to do the pap smear, and I’m like, so what next? Like? What do I expect? And all she has to say is, well, we wait for the results, and then I’ll tell you what to do, and I’m well. Give me a little bit more information than that. You know what I mean like. I have no idea what’s going on. I am very uncomfortable doing this in the 1st place, so I would love a little bit more context.*” (P09). These sentiments suggest that for HPV-SS to be trusted and widely accepted, concerns about test features, instructions, and follow-up support must be addressed through improved education and design.

### 3.4. Suggestions for HPV-SS Implementation

Participants offered a wide range of suggestions to improve the accessibility, safety, and emotional acceptability of HPV-SS. These insights are described across three sub-themes: Distribution and Delivery Preferences, Communication and Education, and Equity and Inclusion.

#### 3.4.1. Distribution and Delivery Preferences

Participants strongly supported flexible and accessible models for the distribution of HPV-SS kits. Multiple delivery channels were proposed. Many participants supported distribution via pharmacies, doctor’s offices, and sexual health clinics. Several participants described that they would like to see the HPV-SS kit more widely accessible: “*I would like to see it available at Shoppers Drug Mart for free, just like the Naloxone kits.*” (P04). One participant expressed the need for widespread implementation, rather than only distributing the kit in clinical settings: “*I would like it to be implemented in community too, yeah. In pharmacies. Because when it is only in the healthcare centers and the hospitals… it’s gonna take time for us to access it.*” (P17). Community hubs, libraries, churches, supermarkets, and convenience stores were also mentioned as potential spaces to access HPV-SS kits: “*Supermarkets, because that’s where you’ll find people—and youth, especially in summer.*” (P08). Although HPV-SS is recommended for those with a cervix who are older than 25 years old, many participants expressed the desire to make HPV-SS kits accessible in youth-oriented spaces such as schools and guidance counselor offices.

Mail was also described as ideal by many participants. Some participants noted that mailed kits would be appropriate only if people could opt in (rather than being sent the kit at random), in order to respect individual privacy and avoid exacerbating stigma: “*Where, like…if it were just sent to me in the mail…Like…And I didn’t know that was coming I would have some issues with that, I think, but mostly out of just like privacy. Things like not all of my living situations have been ones where I would even want to admit being sexually active.*” (P03). Systems like “automatic refills” at pharmacies were described as having potential to be translated into HPV-SS implementation: “*Yeah, I think the idea of being able to sign up so that you’ve got it in the mail like, if that’s what you want. It is actually a really cool idea, because also it would help to remind you to like, you know, do it annually or however often you should be, and if it comes in the mail, it’s like, Oh, right! I should do this, and then you drop it off to your local pharmacy*.” (P30). 

While participants were broadly supportive of the convenience of distribution by mail, several participants voiced concerns about kits being distributed directly by the government, reflecting their anxiety around privacy, stigma, and perceived surveillance: “*Yeah, because it’s the government. And it’d be like, what? Where? What do you? What? Why, what are you doing with this information? Why are you collecting this information? And who’s gonna have access to it? It just immediately makes me think of like an identifying database that I don’t know if they need that.*” (P13). Participants clearly expressed the need to make a government delivery system opt-in: “*If the government… like, didn’t ask me and just started sending me them, I probably wouldn’t take them, you know.*” (P02).

While physical access and distribution were critical, participants also emphasized the need for clear, non-stigmatizing educational tools and communication supports to foster trust and confidence in HPV-SS.

#### 3.4.2. Communication and Education

Many participants described that clear instructions, upfront education, and supportive follow-up pathways are necessary to encourage the use of and engagement with HPV-SS. Accessible and clear instructions, such as representations of how to insert the HPV-SS swab, were described as potentially helpful: “*I think if we could also have maybe a dummy showing the anatomy there…maybe demonstrate like, explain the best position.*” (P24). Foundational knowledge was also described as important, with a few participants suggesting options for sexual health education prior to the delivery of HPV-SS: “*And so just like a little bit of precursor knowledge would be important…it would have been good for me to read it on the way there, so that I would felt more informed about what I was going into.*” (P32). One participant suggested an informational phone line or education at community centers for accessible information on sexual health and screening questions: “*I think on 1st hand, a direct line where you can find information, but directly from a physician… there’s so much information, misinformation as well. There’s a mix of facts that are true, and some others that are not so true. So I think, having a line—whatever that is—phone, email… But also having the option… like a community center where you can go and just like, sit with someone, ask the questions you need to you need to ask and also getting more information as well.*” (P01).

#### 3.4.3. Equity and Inclusion

Several participants recommended gender-inclusive and trauma-informed language across all materials and implementation of HPV-SS. Many participants flagged that current HPV and sexual health education materials are overly focused on women, making other individuals with a cervix feel excluded or misrepresented: “*I didn’t really like how…it only referred to like women doing the test cause, I’m not a woman.*” (P08); “*I’m a trans man with a cervix. The language didn’t include me.*” (P16). Participants described the importance of body-specific language to avoid the exclusion of those that are eligible for HPV-SS and should access sexual health information: “*NOT centering the language around women, or like being female, necessarily just on like the importance of if you have a cervix, you are at risk for cervical cancer.*” (P10). For neurodivergent and marginalized populations, one participant described the importance of direct and clear language and terminology, especially when describing the test: “*Knowing that to the people who are using the swab had sex like using clear language, I think we’re back to language is really important, because, especially being a sex worker like insertion and penetration like, I know what these words mean. And actually, I feel very happy as an autistic person when someone’s very clear about. Yes, insertion.*” (P11). The same participant also expressed that safety and an understanding of what it means to be marginalized are an important component of implementing this type of service: “*So yeah, that’s kind of like just making it safe and seen and knowing that whatever marginalized version of a person you are, you can be, and being a sex worker is marginalized, being an a trans person is marginalized. So knowing that like, how can we make these people feel safe when, no matter what they’ve…99.99% If not, everybody has experienced sexual trauma in some way. That’s on the patriarchy.*” (P11)

Participants emphasized the importance of inclusive design, both in language and delivery, to ensure that HPV-SS initiatives are accessible to people across gender identities, neurodiversity, and lived experiences of trauma and marginalization.

## 4. Discussion

Our study explored the experiences of sex workers and formerly incarcerated people with a cervix regarding the challenges they faced in undertaking cervical cancer screening through Pap tests and HPV self-sampling (HPV-SS) as an alternative screening method. The study was conducted prior to March 2025, when HPV testing replaced the Pap test as the primary method of cervical cancer screening in Ontario, where the study took place. Although HPV-SS was not included in the new screening guidelines, participants’ experiences mainly centered around undergoing Pap tests, which involve visiting a healthcare provider and using a speculum to collect a specimen for testing abnormal cervical cells. This process remains similar under the updated guidelines, where a healthcare provider still uses a speculum to collect a sample, now tested for high-risk HPV.

The findings highlighted significant barriers to traditional cervical cancer screening while also identifying HPV self-sampling (HPV-SS) as a promising and empowering alternative. Participants’ narratives illustrated how personal trauma, systemic failures within the healthcare system, and pervasive societal stigma intersected to hinder access to life-saving care. Additionally, participants offered thoughtful recommendations for strengthening HPV-SS programs to more effectively address the needs of marginalized populations.

It is important to interpret this study’s findings in light of several limitations. First, the findings may not fully represent the experiences of sex workers and formerly incarcerated people who did not frequent our two collaborating agencies, where we identified peer ambassadors—trusted members of the community—who assisted with participant recruitment. As a result, the findings may not be generalizable to the broader community of sex workers and formerly incarcerated people with a cervix, particularly those living outside urban areas. Second, participants who agreed to take part in the focus groups—comprising approximately 40% of our original sample—may have had different experiences or attitudes toward HPV self-sampling (HPV-SS) compared to those who declined participation, potentially introducing selection bias. Third, the presence and facilitation style of the focus group moderator may have influenced the discussions, particularly if participants felt constrained in expressing dissenting or negative views. However, reflections on the narratives shared suggest that participants felt safe and comfortable expressing their perspectives. Additionally, we did not collect data on whether participants identified as sex workers, formerly incarcerated, or both. While this protected participant confidentiality, it limited our ability to further analyze data by identity. Finally, the study was conducted exclusively in English, thereby excluding individuals with limited English proficiency who may face additional, unvoiced barriers to cervical cancer screening. A larger-scale study is needed to fully capture the experiences and challenges faced by the broader community and to ensure greater inclusivity across gender identities, ethnic backgrounds, and geographic locations.

Our findings corroborated earlier research suggesting that traditional cervical cancer screening methods (i.e., Pap tests) for our study communities are often intertwined with fear, stigma, and systemic exclusion [23,24,25]. Participants’ narratives about encounters with disrespectful, judgmental, or fear-inducing healthcare providers not only discouraged them from seeking timely screening but also reinforced long-standing trauma associated with their identities as sex workers, formerly incarcerated individuals, and gender-diverse people [23,24,25].

The lack of accessible and relevant sexual health education further exacerbated these challenges. Participants’ limited access to clear, non-judgmental information impeded their ability to make informed decisions about their health, underscoring the urgent need for targeted and inclusive educational interventions. Structural barriers—including long wait times, inaccessible healthcare settings, and logistical constraints such as transportation—further compounded disparities in cervical cancer screening. These findings highlight the importance of co-developing sexual health education programs that address the specific learning needs of affected communities. In particular, consideration must be given to literacy levels, neurodivergent ways of processing information, and the limitations of “cookie-cutter” educational materials traditionally provided by healthcare providers or organizations such as Cancer Care Ontario. Current sexual health materials often fail to fully accommodate the diverse cultural, sexual, and cognitive realities of individuals with a cervix and are rarely delivered in a trauma-informed manner [26]. Thus, there is a critical need to co-develop culturally sensitive, sex work-affirming, trauma-informed, and gender-affirming sexual health educational resources in collaboration with affected communities, focusing on cervical cancer and HPV self-sampling (HPV-SS). It is essential to meaningfully include neurodivergent individuals in the development process, recognizing that standard educational approaches may not meet their diverse cognitive and communication needs [27]. Materials should be available in multiple languages and accessible formats (e.g., plain language, audio, visual) and should be designed to accommodate different learning styles and processing abilities to ensure truly inclusive access to information.

Despite the profound challenges faced by sex workers and formerly incarcerated people with a cervix in accessing healthcare in general, and cervical cancer screening in particular, participants overwhelmingly viewed HPV self-sampling (HPV-SS) as a transformative option. HPV-SS was described as empowering, offering autonomy, privacy, and a means to reclaim control over one’s body and healthcare decisions. It enabled participants to restore their dignity and exercise their reproductive rights. The self-directed nature of the test significantly mitigated fears of medical trauma and stigma, providing participants with a rare opportunity to engage with screening on their own terms. In particular, participants emphasized that avoiding the use of a speculum—a standard but invasive part of Pap testing—helped them to avoid retraumatization associated with past experiences of sexual abuse or assault. Additionally, the ease of use, minimal invasiveness, and ability to avoid potentially retraumatizing clinical encounters were cited as major benefits. These findings are consistent with previous research suggesting that HPV self-sampling can significantly improve screening uptake among under-screened and marginalized populations [27,28].

However, concerns about the fragility of the swab device and the need for clearer follow-up protocols underscore the importance of not only offering self-sampling but also ensuring that comprehensive support structures are in place. Furthermore, it is crucial to provide HPV self-sampling (HPV-SS) widely and discreetly through non-clinical avenues, such as pharmacies, community centers, and harm reduction sites, where individuals can access them confidentially, as emphasized by our study participants. It is not just about conducting the test, but also about ensuring that individuals are supported through the emotional and psychological aspects of the process, particularly when the test result is positive. Many participants expressed fear that a positive result might equate to a death sentence. This highlights the urgent need for psychological support during the waiting period and after receiving the results. Establishing 24/7 helplines or telehealth services, where trained nurses or psychologists are available to provide support, is essential. These professionals can offer education on the meaning of the results in a simple, easy-to-understand, and non-judgmental manner, and connect individuals to appropriate follow-up care when necessary. Additionally, it is crucial to provide training for healthcare providers in trauma-informed, anti-stigma care. The lack of such training presents a significant barrier to accessing equitable care [29]. Providers should be equipped to counter biases against sex workers, queer and trans individuals, and those with incarceration histories, ensuring that healthcare spaces are safe, inclusive, and welcoming for all [30].

By centering the voices of structurally marginalized populations and designing interventions that prioritize autonomy, dignity, and accessibility, we can move closer to achieving equitable cervical cancer screening outcomes.

## 5. Conclusions

This qualitative study provides valuable insights into the barriers and facilitators surrounding cervical cancer screening and HPV self-sampling (HPV-SS) among sex workers and formerly incarcerated people. Participants highlighted a range of structural, interpersonal, and emotional factors that hindered access to conventional cervical cancer screening, including healthcare-related stigma, past traumatic experiences, and limited access to information. In this context, HPV-SS was widely viewed as a more acceptable and accessible alternative, valued for its privacy, autonomy, and ease of use.

Despite broad support for HPV-SS, participants emphasized several critical implementation needs. These included clear, accessible instructions; transparent and supportive follow-up procedures for individuals with positive results; and delivery models that safeguard privacy and respect individual choice. Opt-in systems, pharmacy-based access, and community-centered distribution were identified as key facilitators. Additionally, participants strongly advocated for the use of gender-inclusive and trauma-informed language in all educational materials and communications.

These findings underscore the necessity of embedding HPV-SS programs within care systems that are responsive to the specific needs of marginalized populations. Addressing structural inequities, using inclusive language, and implementing community-based outreach will be essential to ensuring that HPV-SS serves as a meaningful tool in the prevention of cervical cancer among underserved groups.

## Data Availability

Participants of this study did not give written consent for their data to be shared publicly, so due to the sensitive nature of the research, supporting data is not available.

## Abbreviations

The following abbreviations are used in this manuscript:

HPV-SS	HPV self-sampling
GTA	Greater Toronto Area

## References

[1] Duff P., Ogilvie G., Shoveller J., Amram O., Chettiar J., Nguyen P., Dobrer S., Montaner J., Shannon K. (2016). Barriers to cervical screening among sex workers in Vancouver. Am. J. Public Health.

[2] Benoit C., Ouellet N., Jansson M. (2016). Unmet health care needs among sex workers in five census metropolitan areas of Canada. Can. J. Public Health.

[3] Kouyoumdjian F.G., McConnon A., Herrington E.R., Fung K., Lofters A., Hwang S.W. (2018). Cervical cancer screening access for women who experience imprisonment in Ontario, Canada. JAMA Netw. Open.

[4] Di Giuseppe G., Folcarelli L., Lanzano R., Napolitano F., Pavia M. (2022). HPV vaccination and cervical cancer screening: Assessing awareness, attitudes, and adherence in detained women. Vaccines.

[5] Kelly P.J., Allison M., Ramaswamy M. (2018). Cervical cancer screening among incarcerated women. PLoS ONE.

[6] Ojerinde A.C., Thorne S.E., Howard A.F., Kazanjian A. (2024). Cervical Cancer Screening Uptake and Experiences of Black African Immigrant Women in Canada. Glob. Qual. Nurs. Res..

[7] Benjamin K.A., Lamberti N., Cooke M. (2023). Predictors of non-adherence to cervical cancer screening among immigrant women in Ontario, Canada. Prev. Med. Rep..

[8] Wong J., Vahabi M., Miholjcic J., Tan V., Owino M., Li A., Poon M. (2018). Knowledge of HPV/cervical cancer and acceptability of HPV self-sampling among women living with HIV: A scoping review. Curr. Oncol..

[9] Yang H., Letendre A., Shea-Budgell M., Bill L., Healy B.A., Shewchuk B., Nelson G., Newsome J., Chiang B., Rahul C.R. (2024). Cervical cancer screening outcomes among First Nations and non-First Nations women in Alberta, Canada. Cancer Epidemiol..

[10] McDonald J.T., Kennedy S. (2007). Cervical cancer screening by immigrant and minority women in Canada. J. Immigr. Minor. Health.

[11] Ross L.E., Sterling A., Dobinson C., Logie C.H., D’Souza S. (2021). Access to sexual and reproductive health care among young adult sex workers in Toronto, Ontario: A mixed-methods study. Can. Med. Assoc. Open Access J..

[12] Cervical Cancer Testing and Prevention|Ontario.ca [Internet]. https://www.ontario.ca/page/cervical-cancer-testing-and-prevention.

[13] Arbyn M., Smith S.B., Temin S., Sultana F., Castle P. (2018). Detecting cervical precancer and reaching underscreened women by using HPV testing on self samples: Updated meta-analyses. BMJ.

[14] Arbyn M., Castle P.E., Schiffman M., Wentzensen N., Heckman-Stoddard B., Sahasrabuddhe V.V. (2022). Meta-analysis of agreement/concordance statistics in studies comparing self-vs clinician-collected samples for HPV testing in cervical cancer screening. Int. J. Cancer.

[15] Ogilvie G.S., Patrick D.M., Schulzer M., Sellors J.W., Petric M., Chambers K., White R., FitzGerald J.M. (2005). Diagnostic accuracy of self collected vaginal specimens for human papillomavirus compared to clinician collected human papillomavirus specimens: A meta-analysis. Sex. Transm. Infect..

[16] World Health Organization (2021). WHO Guideline for Screening and Treatment of Cervical Pre-Cancer Lesions for Cervical Cancer Prevention: Use of mRNA Tests for Human Papillomavirus (HPV).

[17] Ogilvie G., Krajden M., Maginley J., Isaac-Renton J., Hislop G., Elwood-Martin R., Sherlock C., Taylor D., Rekart M. (2007). Feasibility of self-collection of specimens for human papillomavirus testing in hard-to-reach women. CMAJ.

[18] Lofters A., Devotta K., Prakash V., Vahabi M. (2021). Understanding the Acceptability and Uptake of HPV Self-Sampling Amongst Women Under- or Never-Screened for Cervical Cancer in Toronto (Ontario, Canada): An Intervention Study Protocol. Int. J. Environ. Res. Public Health.

[19] Devotta K., Vahabi M., Prakash V., Lofters A.K. (2023). Implementation of a Cervical Cancer Screening Intervention for Under- or Never-Screened Women in Ontario, Canada: Understanding the Acceptability of HPV Self-Sampling. Curr. Oncol..

[20] ICO/IARC Information Center on HPV and Cancer Canada: Human Papillomavirus and Related Cancer, Fact Sheet 2023. https://hpvcentre.net/statistics/reports/CAN_FS.pdf.

[21] Ackerson K. (2012). A history of interpersonal trauma and the gynecological exam. Qual. Health Res..

[22] Vahabi M., Hynes J., Wong J.P., Kithulegoda N., Moosapoor M., Akbarian A., Lofters A. (2024). Breaking Barriers: Empowering Cervical Cancer Screening with HPV Self-Sampling for Sex Workers and Formerly Incarcerated Women in Toronto. Curr. Oncol..

[23] Fuzzell L.N., Perkins R.B., Christy S.M., Lake P.W., Vadaparampil S.T. (2021). Cervical cancer screening in the United States: Challenges and potential solutions for underscreened groups. Prev. Med..

[24] Milner G.E., McNally R.J. (2020). Nonadherence to breast and cervical cancer screening among sexual minority women: Do stigma-related psychological barriers play a role?. Health Psychol..

[25] Driscoll S.D. (2016). Barriers and facilitators to cervical cancer screening in high incidence populations: A synthesis of qualitative evidence. Women Health.

[26] Larki M., Taffazoli M., Latifnejad Roudsari R., Babaee A. (2015). The impact of an educational program on knowledge and attitude of female sex workers in preventing high risk sexual behaviours. J. Midwifery Reprod. Health.

[27] Gillibrand S., Gibson H., Howells K., Urwin S., Davies J.C., Crosbie E.J., Sanders C. (2025). Exploring the barriers to cervical screening and perspectives on new self-sampling methods amongst under-served groups. BMC Health Serv. Res..

[28] Daponte N., Valasoulis G., Michail G., Magaliou I., Daponte A.I., Garas A., Grivea I., Bogdanos D.P., Daponte A. (2023). HPV-based self-sampling in cervical cancer screening: An updated review of the current evidence in the literature. Cancers.

[29] Marshall D.C., Carney L.M., Hsieh K., Dickstein D.R., Downes M., Chaudhari A., McVorran S., Montgomery G.H., Schnur J.B. (2023). Effects of trauma history on cancer-related screening, diagnosis, and treatment. Lancet Oncol..

[30] Squires K. (2024). Sex Workers in Canada Face Unequal Access to Healthcare: A Systems Thinking Approach. J. Prim. Care Commun. Health.

